# The Role of Amodal Surface Completion in Stereoscopic Transparency

**DOI:** 10.3389/fpsyg.2012.00351

**Published:** 2012-09-17

**Authors:** Barton L. Anderson, Alexandra C. Schmid

**Affiliations:** ^1^School of Psychology, University of SydneySydney, NSW, Australia

**Keywords:** stereopsis, scission, transparency, occlusion, lightness, depth perception, color vision

## Abstract

Previous work has shown that the visual system can decompose stereoscopic textures into percepts of inhomogeneous transparency. We investigate whether this form of layered image decomposition is shaped by constraints on amodal surface completion. We report a series of experiments that demonstrate that stereoscopic depth differences are easier to discriminate when the stereo images generate a coherent percept of surface color, than when images require amodally integrating a series of color changes into a coherent surface. Our results provide further evidence for the intimate link between the segmentation processes that occur in conditions of transparency and occlusion, and the interpolation processes involved in the formation of amodally completed surfaces.

## Introduction

There is an extensive and growing body of work on how the visual system segments images into a stratified representation of surfaces in depth. Some of the most compelling examples of this form of image segmentation arise from conditions of occlusion and transparency. Kanisza (1979) noted the similarity in the perceptual organization that occurs in conditions of occlusion and transparency, which involve an awareness of a “double presence” of surfaces along the same line of sight. The view of underlying surfaces is hidden completely by occluding surfaces, but is nonetheless experienced to continue or complete amodally behind the occluder. In conditions of transparency, the near surface only partially obscures the underlying surface, and both the transparency and underlying surface are simultaneously visible along the same line of sight. The commonality in the perceptual organization of transparency and occlusion suggests that they reflect the outputs of a common set of computations or processes (Anderson and Julesz, 1995; Anderson, 1997, 2003a, 2007; Anderson et al., 2002).

**Figure 1:**
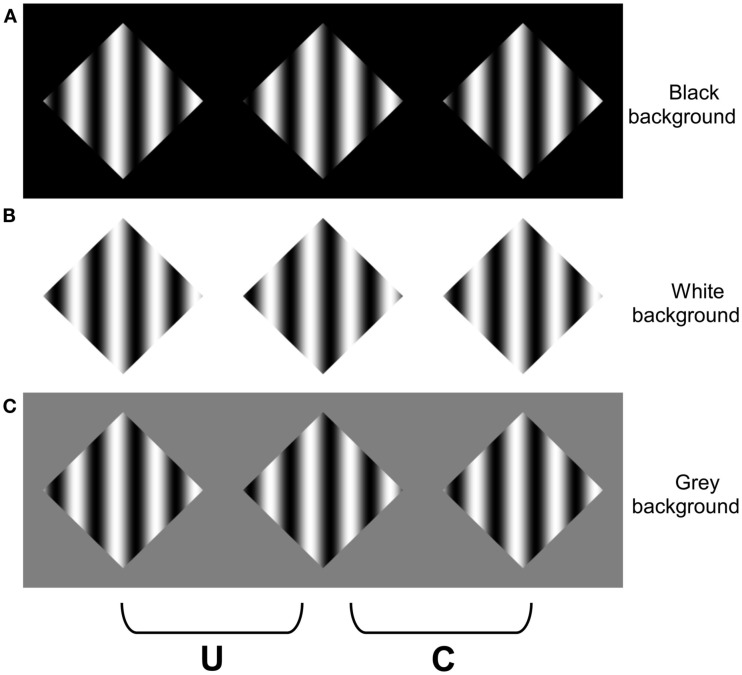
**Stereoscopic grating stimuli**. For these and all subsequent stereograms, three images are presented so that the effects of placing the texture in front of the aperture boundary can be experienced with either crossed fusion (marked C on each figure) or uncrossed (divergent) fusion (marked U on each figure). Fusing the other two images places the grating (texture) behind the aperture boundary. When the grating is given a far disparity relative to the aperture boundary, it appears behind the aperture edges and surround (any perceived depth within the grating arises from the interpretation of the gradients as surface shading, not from stereopsis). However, when the disparity of the boundary is inverted, the stereograms in **(A,B)** appear to split into two layers: hazy black bars occluding a white diamond in **(A)**, and hazy white bars occluding a black diamond in **(B)**. No coherent percept of scission is experienced within the grating for the gray background in **(C)**. Note that the perception of occlusion and transparency in **(A,B)** arise from a combination of segmentation processes that split the grating into two components, and integration processes that amodally integrate the disparities along the diamond boundary into a coherent surface. Adapted from Anderson (1999), with permission.

**Figure 2:**
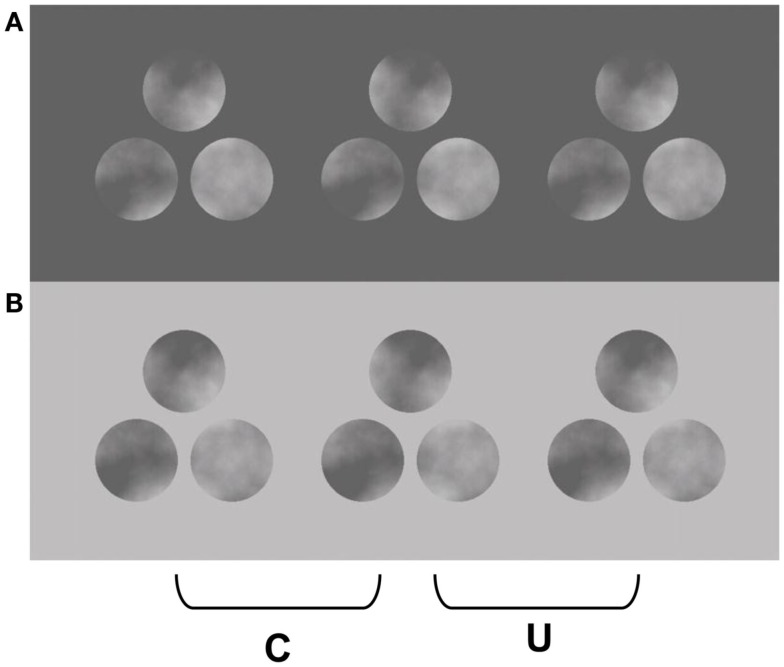
**Two-dimensional noise analogues of the stereo images presented in Figure 1**. When the two left panels are cross fused or the two right panels divergently fused, the texture appears to split into two layers: dark clouds over light disks in **(A)**, and light clouds over dark disks in **(B)**. Note that the lightest regions within the texture on the dark surround appear as portions of a far surface in plain view, and similarly for the darkest regions of the texture on the light surround. Adapted from Anderson (1999), with permission.

The link between occlusion and transparency has been particularly evident in studies where textured targets are decomposed into percepts of inhomogeneous transparency (Anderson, 1999, 2003a,b, 2007; Anderson and Winawer, 2005, 2008; Anderson et al., 2006). In appropriate geometric and photometric conditions, a texture containing continuous luminance modulations can appear to perceptually split (or *scission*) into multiple layers. The variations in luminance within the texture are perceived as fluctuations in the opacity of a transparent surface, which appear to vary smoothly between completely opaque to fully transparent. Examples of stereoscopic versions of these displays are presented in Figures 1 and 2, which depict 1D and 2D luminance modulations, respectively (cf. Anderson, 1999). The textures are placed within apertures on a homogenously colored surround, and disparity is introduced by horizontally shifting the boundaries of the apertures relative to the textures in the two eyes (see Figures 1 and 2). When the texture’s disparity places it behind the aperture, the texture simply appears as a flat, opaque surface, as would be predicted on the basis of the disparities present. But when the relative disparities are inverted (by swapping the two eyes’ images), and the texture has a disparity that should place it in front of the surround, the texture can appear to split into multiple layers: A near, inhomogeneous transparent surface that appears to vary in perceived opacity; and a more distant (opaque) surface that appears uniformly colored. One of the remarkable aspects of this percept is that there are no disparities within the texture that specify an underlying surface; the only disparity at this more distant depth occurs along the aperture boundary. The particular pattern of perceived transparency experienced, and the perceived lightness of the two layers, depends critically on the luminance relationships with the surround. When the surround is as bright (or brighter) than the brightest luminance modulations within the texture, the transparent layer also appears light, and the far layer appears to be uniformly dark (Figures 1A and 2A). When the surround is as dark (or darker) than the darkest luminance modulations within the texture, the transparent layer appears dark, and the far layer appears to be uniformly light (Figures 1B and 2B). Critically, however, when the surround color falls between the luminance values of the texture, no coherent percept of transparency is experienced throughout the grating (Figure 1C). Some piece-wise percepts of transparency can be achieved, but this display does not give rise to a coherent percept of scission as that experienced in Figures 1A,B.

The percepts that are experienced when fusing Figures 1 and 2 demonstrate the strong interplay between photometric and geometric (depth) constraints on the perception of transparency. Metelli (1974a,b) originally articulated some of the relevant photometric constraints for “balanced” transparent surfaces (i.e., transparent surfaces with a homogeneous reflectance and transmittance), which were subsequently abstracted and generalized to inhomogeneous forms of transparency (Anderson, 1999, 2003a; Anderson and Winawer, 2005, 2008; Anderson et al., 2006). A strong cue to the presence of transparency is the contrast polarity (or sign) of contours and textures: transparent surfaces only alter the contrast magnitudes of underlying textures and contours, they do not alter their polarity (Beck et al., 1984; Beck, 1986; Beck and Ivry, 1988; Adelson and Anandan, 1990; Gerbino, 1994; Anderson, 1997). This simple polarity constraint can provide some understanding of why the textured targets in Figures 1 and 2 on the dark and light surrounds support the percept of transparency, but the mid-gray surround in Figure 1C does not: The more distant texture-surround contour has a consistent contrast polarity in the light and dark surrounds, but the gray surround causes texture-surround boundary to undergo numerous contrast polarity reversals. Moreover, the contrast reductions that occur along the surround-texture boundary in the polarity preserving surrounds are consistent with variations in the opacity of a transparent layer, which is how they are perceived.

The interplay between geometric and photometric constraints in determining perceived lightness is somewhat more complex, and has been described at length previously (Anderson et al., 2002; Anderson, 2003a, 2007). One constraint arises from the geometry of occlusion, which was termed a contrast depth asymmetry principle (CDAP). The CDAP was articulated to answer the following question: given a local luminance difference (i.e., local contrast signal), with a particular depth (here, disparity), what can be concluded about the depth and relative brightness of the luminance *components* that generated this signal? The answer provided by the CDAP can be best appreciated by considering a simple luminance discontinuity (“edge”), say, a vertical edge that is light on its left and dark on its right (see Anderson et al., 2002; Anderson, 2003a). An edge of this kind can be generated in the image in one of four ways: (1) a change in surface pigmentation or illumination lying along a continuous surface; (2) a 3D fold or shape discontinuity which meet to form the edge; (3) one of two types of occluding contour (light occluding dark, or dark occluding light), or (4) a higher contrast edge of any of the above kinds overlaid with a (contrast reducing) transparent layer. All of these possibilities require that luminance is mapped onto depth in such a way that the light and dark sides of the edge appear at the depth of the edge, or in the case of occlusion, one side of the edge can appear more distant. Note, however, that because transparent overlays can cause a reduction in the contrast of underlying lightness differences, the CDAP only prescribes how *ordinal* brightness relationships are constrained by the depth and polarity of local contrast signals. It is always possible that the contrast of an edge has been reduced by a transparent overlay, and hence that the local luminance difference in the image underestimates the luminance difference in the world. This implies the photometric constraint imposed by the CDAP only applies to the *polarity* or local contrast signals, not their magnitudes. A second constraint – a transmittance anchoring principle (TAP) – is needed to explain whether a local image contrast is seen in plain view or through a transparent layer (Anderson, 2003a). This principle states that the visual system has a bias to interpret the highest contrast segments of contours and surfaces as regions in plain view, which serve as anchor points for scaling the perceived opacity of transparent layers (Anderson et al., 2002, 2006; Anderson, 2003a).

Consider the role of the CDAP and TAP in shaping the percepts observed in Figures 1 and 2. When the disparity of the aperture boundary is more distant than the disparity of the texture, both the light and dark sides of the aperture boundary must appear at this more distant depth, at least in the immediate neighborhood of this boundary. Thus, the regions adjacent to the dark surround within the textures must appear light, and the regions adjacent to the light surround must appear dark. The highest contrast segments of these contours appear in plain view, such that the “white” regions in the grating on the dark surround appear as portions of the diamond figure in plain view, and similarly for the “dark” regions in the grating on the light surround. The TAP states that this highest contrast contour segment serves as an anchor point in which the variations in contrast strength that occur along the aperture boundaries in Figures 1A,B and 2A,B are interpreted as variations in the opacity of transparent layers. Note that as the contrast between the surround and the texture elements decreases, the perception of opacity of the transparent layer increases, and conversely (i.e., the dark regions within the texture on the dark surround appear most opaque, and the light regions of the texture appear most opaque on the light surround). Note that the global percept of scission in these images results from a propagation of these constraints from along the far contour to the interior of the grating texture (as well as the 2D textures depicted in Figure 2).

**Figure 3:**
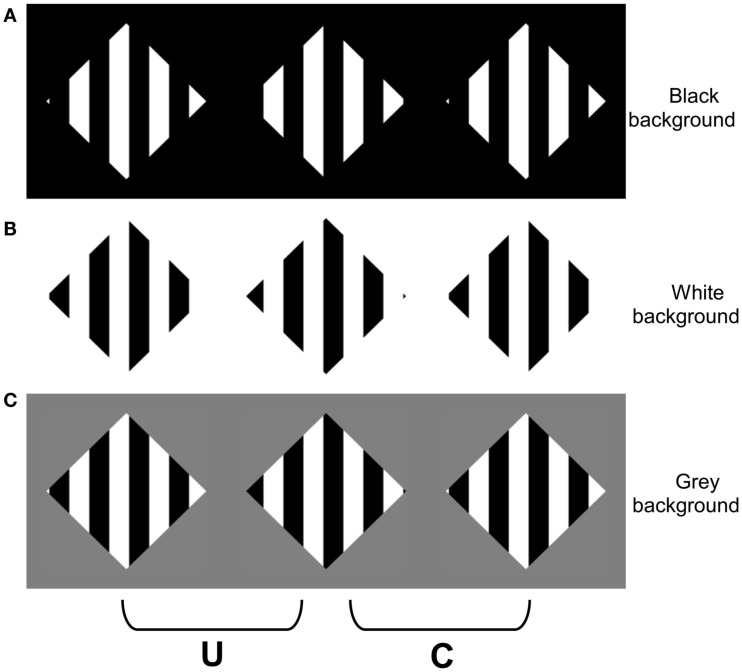
**Square-wave versions of the stimuli in Figure 1**. When the two right columns are cross fused (or two left fused divergently), **(A)** appears as a white diamond occluded by black stripes, **(B)** appears as a black diamond occluded by white stripes, and **(C)** appears incoherent.

A similar analysis can explain the failure to experience a coherent percept of transparency when the surround luminance lies between the luminance variations within the texture (Figure 1C). The surround-texture aperture boundary in this display contains numerous polarity reversals. When the aperture boundary is behind the texture, the CDAP requires the textured regions adjacent to the aperture boundary to alternate between dark and light, depending on the polarity of the aperture edge. However, this information conflicts with the disparity signals that are generated *within* the texture, which place all of the contrast changes of the texture in the near depth plane. This conflict can be appreciated by considering a simplified square-wave version of this stimulus presented in Figure 3. The stereograms in Figures 3A,B generate simple percepts of occlusion: black stripes occluding a white diamond in Figure 3A, and white stripes occluding a black diamond in Figure 3B. Note that the disparities generated by the vertical contours in the two displays are identical, but the disparities generated by the far diagonal contours (forming the diamond) appear in different locations in the two images (adjacent to the white regions in Figure 3A, and the black regions in Figure 3B), which cause the white regions in Figure 3A, and the black regions in Figure 3B, to appear in the far depth plane. However, when the surround is gray, there are disparities generated by the far diagonal contours adjacent to *both* the black and the white bars. This means that there must be both black and white surfaces at the far depth plane adjacent to the aperture boundary. However, the disparities within the aperture specify that the black stripes, white stripes, or both should appear in *front* of the surround. There is no coherent surface interpretation that can satisfy both of these constraints, which generates significant visual conflict, particularly in regions of the image where the vertical and diagonal contours intersect (i.e., at the T-junctions in the image).

Although there is no coherent surface interpretation that can account for the pattern of disparities within the grating and along the aperture boundaries in the simple occlusion displays depicted in Figure 3C, there is a possible (albeit improbable) interpretation of the related sinusoidal display depicted in Figure 1C. The *gray* regions within the sinusoidal modulation could appear in the near plane (consistent with their disparity), and both the black (luminance minima) and white (luminance maxima) regions of the sinusoid could appear in the far depth plane. This interpretation requires the near, gray regions within the sinusoid to be arranged such that they occlude all of the transitions between the black and white stripes that would otherwise be visible in the far depth surface (i.e., within the diamond). To date, the incoherence of displays such as Figure 1C has been attributed solely to the violations in the luminance polarity constraints on transparency (Anderson, 2003a). However, it is also possible that the incoherence experienced when fusing Figure 1C arises, at least in part, from the difficulty in amodally completing a surface that involves binding surface regions that undergo repeated color changes (i.e., amodally linking black surface regions to white surface regions). The purpose of the experiments reported herein was to test this hypothesis.

## Assessing the Role of Amodal Surface Completion

**Figure 4:**
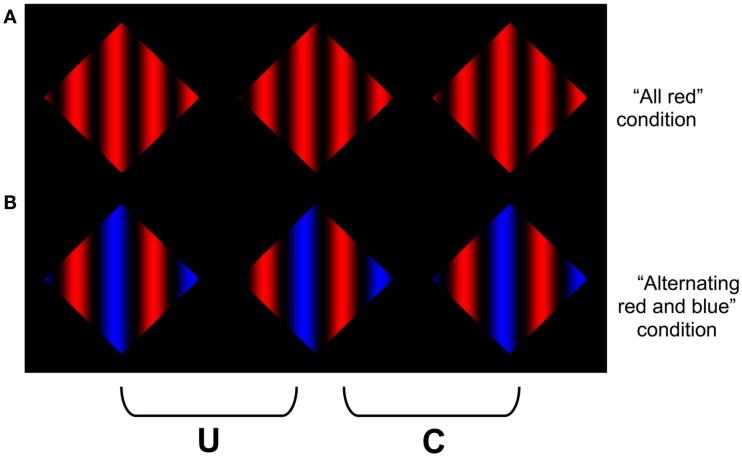
**Examples of the chromatic grating images used in Experiment 1**. The stimuli were constructed by sinusoidally modulating the color of the grating between “black” to “red” in **(A)**, and alternating cycles of red-black and blue-black in **(B)**. Note that it is more difficult to obtain a clear percept of an amodally completed surface in **(B)**. To avoid the modal completion of the dark “components” of the grating across the surround, the reader is advised to occlude the other images in the figure when viewing each row of the figure.

We constructed colored variants of the stereoscopic images depicted in Figure 1 to assess whether the failure to observe coherent percepts of scission in Figure 1C arose from the fact that any such interpretation would require amodally interpolating a surface composed of alternating colors, which would have to be (accidentally) occluded by the near surface. The advantage of using colored displays is that they allow us to dissociate the luminance constraints on transparency from constraints on amodal completion that might arise from differences or similarities in surface color. To this end, we constructed colored variants of Figure 1A. This allowed us to maintain a consistent luminance polarity between the grating and the surround, while varying the chromatic content of the surfaces that would have to be integrated to generate a coherent percept of scission. We began by constructing two types of surfaces: one in which the sinusoidal variation in color was uniform throughout the display (chromatic analogs of Figures 1A,B), and another where the sinusoidal variations alternated between two different colors (a chromatic analog of Figure 1C). We constructed variants in which the sinusoid varied from black to a specified color (red, blue, or green), such that the (luminance) contrast polarity was preserved along the length of the aperture boundary (Figure 4). We observed that it was perceptually more difficult to obtain a coherent percept of multiple layers in the image containing the alternating blue and red cycles of the sinusoidal pattern, which provides phenomenological support for our hypothesis. We sought to provide evidence for this phenomenological evidence using objective psychophysical methods. We reasoned that if it is more difficult to obtain a coherent percept of scission in the display containing alternating color cycles, then it should be harder to detect depth differences in the these displays (i.e., increment detection thresholds should be higher in the alternating cycle display than the uniform color display.) The goal of Experiment 1 was to test this hypothesis.

## Experiment 1

### Methods

#### Participants

Three observers (AS, PM, and KT) participated in this experiment. All observers had normal or corrected to normal vision and were psychophysically experienced. Two observers (PM and KT) were naive as to the purpose of the experiment, and observer AS is one of the authors.

#### Apparatus and stimuli

For all experiments stimuli were presented on a Dell 2405FPW monitor running at a refresh rate of 60 Hz and with a resolution of 1920 × 1200 pixels, controlled by a Mac Pro computer running Mac OS X 10. Stimulus presentation and data collection were controlled by a Matlab 7.4 (MathWorks) script using the Psychophysics Toolbox (Brainard, 1997). Stimuli were viewed through a stereoscope in a dark room at a viewing distance of 70 cm.

The stimuli were vertical sine wave gratings, which were generated by sinusoidally modulating the X-coordinates of the grating color between a particular color (red or blue), and viewing them through a diamond shaped aperture (Figure 4). The aperture subtended 4.33 arc degrees, and the spatial frequency of the grating was 0.923 cycles/degree. The stimuli were presented stereoscopically, and binocular disparities were introduced by slightly offsetting the phase of the gratings between each eye’s views so that the aperture boundary had a far disparity relative to the grating (i.e., rightward in the left eye, and leftward in the right eye). For the uniformly colored stimulus, the resulting percept was of black bars that vary in opacity floating out in front of a colored diamond-shaped Figure.

Two versions of the sine wave grating were created. In the “all red” condition, the sinusoidal modulations were consistent throughout the image, and oscillated between a maximum value of full red to a minimum value of full black (all color guns off; Figure 4A). In the “alternating red and blue” condition, the chromatic cycles of the grating alternated between red to black, and blue to black (Figure 4B). The luminance of the peak of the blue cycle was 5.081 cd/m^2^ (CIE *xy*Y coordinates *x* = 0.15, *y* = 0.064) and the luminance of the peak of the red cycle was 22.308 cd/m^2^ (CIE *xy*Y coordinates *x* = 0.65, *y* = 0.34). The luminance of the black bars (grating) was 0.310 cd/m^2^. The luminance of the black homogeneous surround adjacent to the diamond apertures was the same as the minima of the gratings (0.310 cd/m^2^).

In each trial, two stereoscopic sine wave gratings were displayed. One of the images (the pedestal) had a fixed disparity of 5.2 arc min between the grating and the aperture boundary. For the other image (pedestal + disparity), the disparity between the aperture boundary and grating was the pedestal plus one of the 10 manipulated disparities (0.325, 0.65, 0.975, 1.3, 1.625, 1.95, 2.275, 2.6, 3.25, and 3.9 arc min).

#### Procedure

A two-alternative forced choice (2AFC) method of constant stimuli was used to obtain psychometric functions for the two display types. The 10 disparities were presented 140 times each, for each of the two color conditions, yielding a total of 2800 trials. The trials were divided into 10 blocks, such that each block contained 280 trials and took approximately 15 min. The two display types appeared randomly within a block of trials. Observers were told they could take a break at any time and not all of the different blocks were completed on the same day.

Before each trial two fixation crosses (13 arc min) were displayed in the locations the stimuli would appear, and remained there until the stimuli were presented. Observers pressed the space bar to initiate each trial, after which the pedestal and the pedestal + disparity images were displayed, one on the top half and one on the bottom half of the screen (replacing the fixation crosses). The two images remained on the screen for 2 s before disappearing and displaying a black screen. The location of the pedestal image and the pedestal + disparity image, the presentation order of the 10 manipulated disparities, and the color condition, were all counterbalanced and randomized across trials.

The observers’ task was to indicate which image (top or bottom) in each trial contained a greater depth difference between the black bars and the colored background. Observers indicated their choice via a keyboard press (up-arrow for the top image, down-arrow for the bottom image). Although the stimuli were only presented for 2 s, observers could take as long as they wanted to respond. Immediately after a response, the fixation crosses reappeared, and remained there until observers pressed the space bar to initiate the next trial.

## Results and Discussion

**Figure 5:**
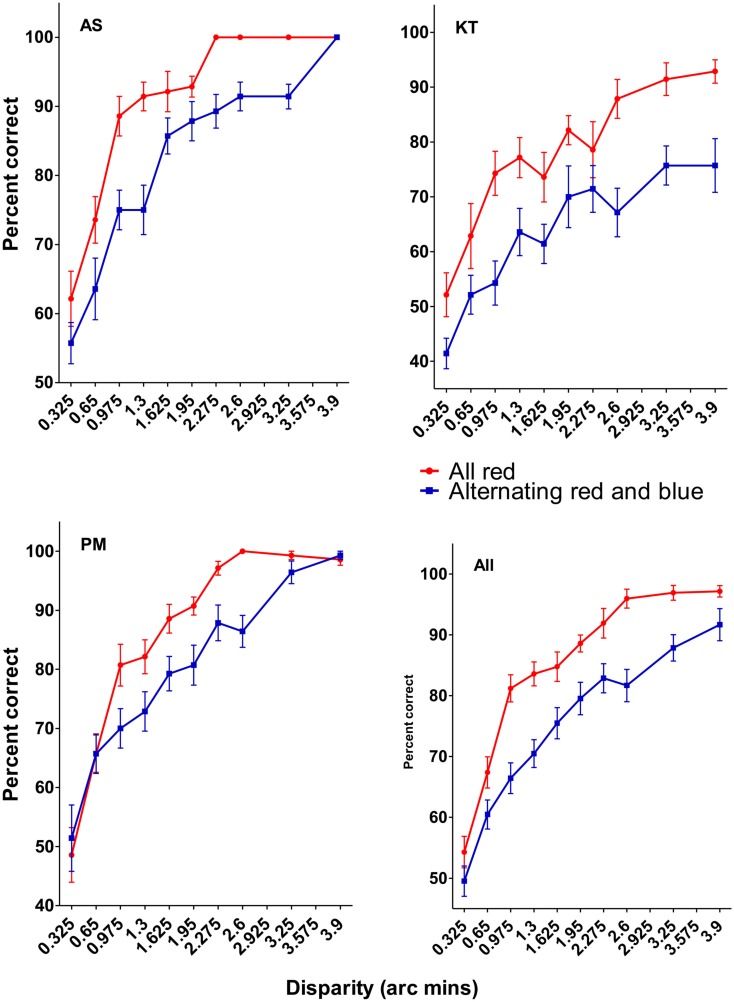
**Results of Experiment 1**. The individual data are plotted in the top two and bottom left figures, and the average data is plotted on the bottom right. Error bars in this and all subsequent figures are SEM. The results show that it was significantly harder discriminating the disparity increment in the mixed color condition than in the uniform color condition.

The results of Experiment 1 are presented in Figure 5. The individual data are plotted in the top two and bottom left panels, and the average data is plotted in the bottom right (for the remainder of the control experiments, only the average data will be plotted). The data reveal that it was harder to detect the depth difference in the display containing the alternating blue and red sinusoidal modulations than the uniformly colored display. In this and subsequent experiments, we used a binomial sign test to compute the likelihood of obtaining *k* or more instances in which the observers’ performance in the alternating display was worse than the uniformly colored display (10 pairs of data points per subject). The results of Experiment 1 are highly significant (*p* < 0.0001), and suggest that the difficulty in perceptually synthesizing a coherent amodally completed surface impaired the ability to detect depth differences in the display containing the alternating red and blue cycles.

There are, however, a number of possible confounds and alternative explanations that could have contributed to this poorer performance. The red and blue colors were arbitrarily chosen because they appear quite different and should be difficult to integrate into a coherent surface. However, these colors are known to give rise to chromo-stereopsis in some observers, which is putatively caused by chromatic aberration. This could cause the red and blue components of the grating to appear at different depths, which could introduce disparity noise in the mixed color condition. Although none of our observers reported any apparent depth differences between the red and blue components of these figures, we performed a control experiment (Experiment 2) to test whether any possible perceived difference in depth contributed to the different pattern of results. We compared the ability of observers to detect a depth difference between an all red-black grating from an all blue-black grating. In one half of the trials, the red stimulus had the smaller (pedestal) disparity; in the other half, the blue stimulus had the smaller (pedestal) disparity. If one of the stimuli was consistently perceived as having a greater depth than the other, then there should be a significant difference in the ability to perform these two discriminations. Specifically, if the display that appears to contain more depth than the other also has the larger physical disparity, then observers should be more accurate in detecting this depth difference than when the opposite condition holds.

## Experiment 2

### Participants

Two observers (AS and KT) who participated in Experiment 1 also participated in experiment 2.

### Apparatus and stimuli

**Figure 6:**
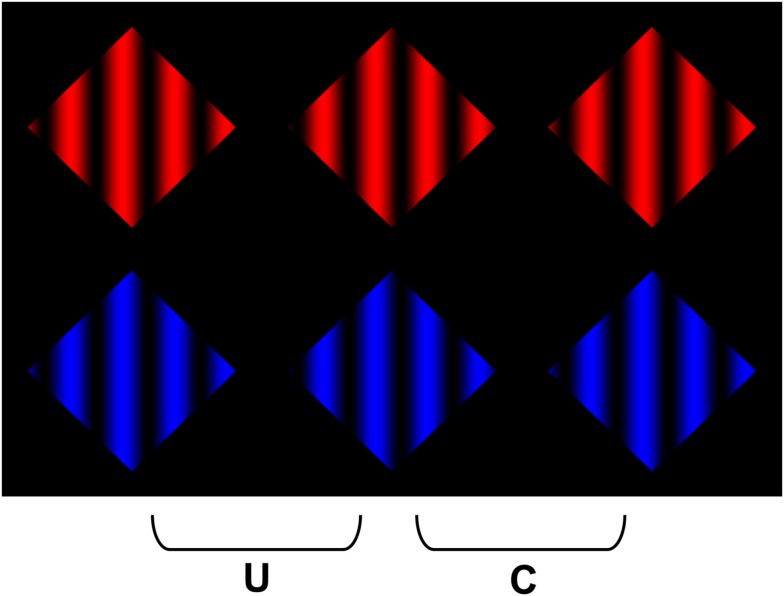
**The stimuli used in Experiment 2 to test for any systematic differences in the perceived depth of the red and blue components of the stereo images used in Experiment 1**.

Stimuli were similar to the “all red” condition used in Experiment 1. Whereas in the Experiment 1 the pedestal and the pedestal + disparity images contained the same colors in a given trial, in Experiment 2 the colors of the two images differed. There were two conditions: In the “blue greater disparity” condition the color of the pedestal image was all red (Figure 6, top) and the color of the pedestal + disparity image was all blue (Figure 6, bottom); in the “red greater disparity” condition the color of the pedestal image was all blue (Figure 6, bottom) and the color of the pedestal + disparity image was all red (Figure 6, top).

### Procedure

The procedure was identical to experiments 1 and 2, except trials were divided into seven blocks instead of ten (420 trials per block).

## Results and Discussion

**Figure 7:**
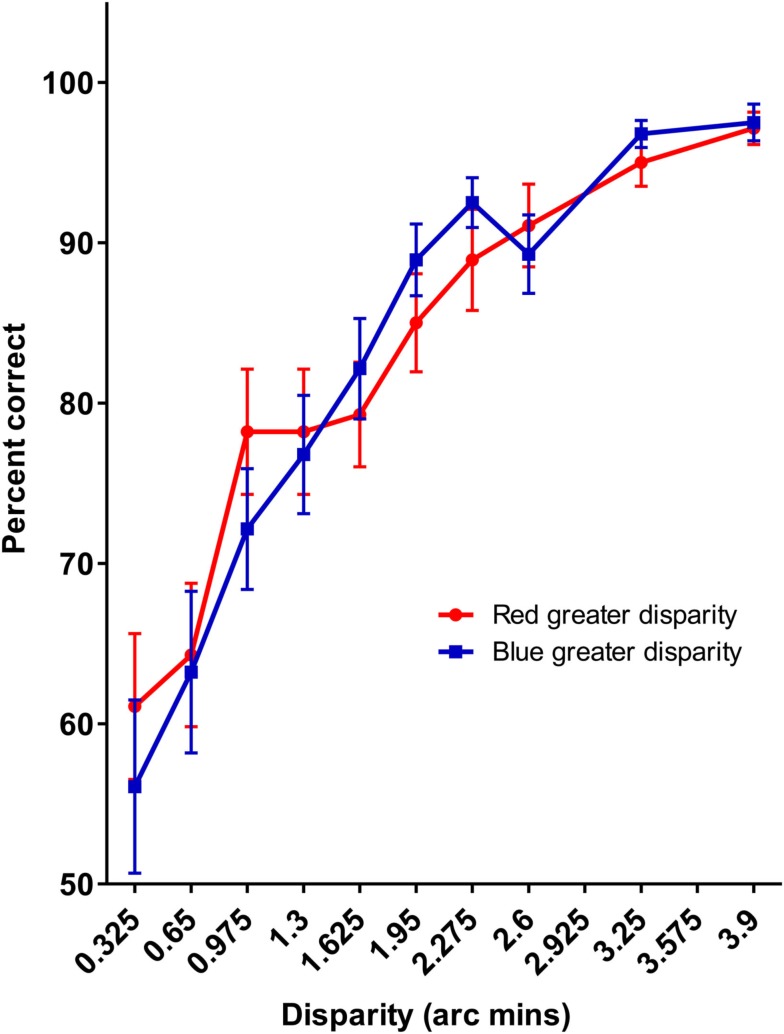
**The averaged results of Experiment 2**. No differences between the red and blue stimuli are observed.

The results of Experiment 2 are presented in Figure 7, averaged over the two observers. No significant differences between the red and the blue pedestal conditions were observed (*p* = 0.588), suggesting that the difficulty in detecting depth in the mixed color displays cannot be attributed to differences in perceived depth between the red and the blue targets.

**Figure 8:**
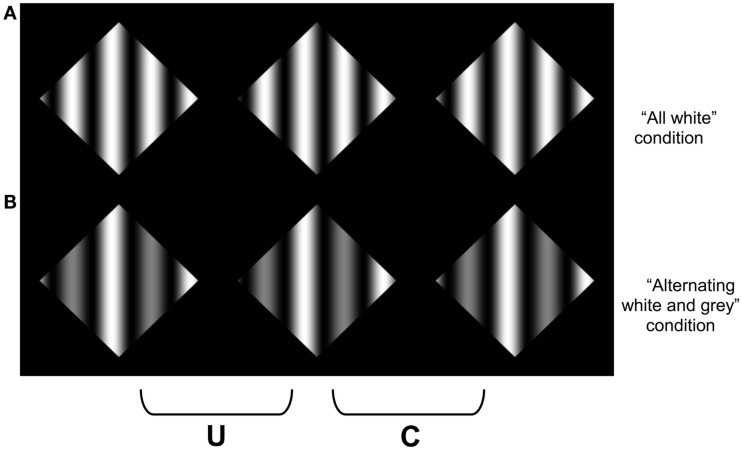
**An amplitude modulated achromatic version of the sinewave stimuli used in Experiment 1**. In **(A)**, all of the cycles of the sinusoid varied from black to white, whereas in **(B)**, alternating cycles varied between black to white, and black to gray. Note that a coherent percept of scission may still be observed in **(B)**.

**Figure 9:**
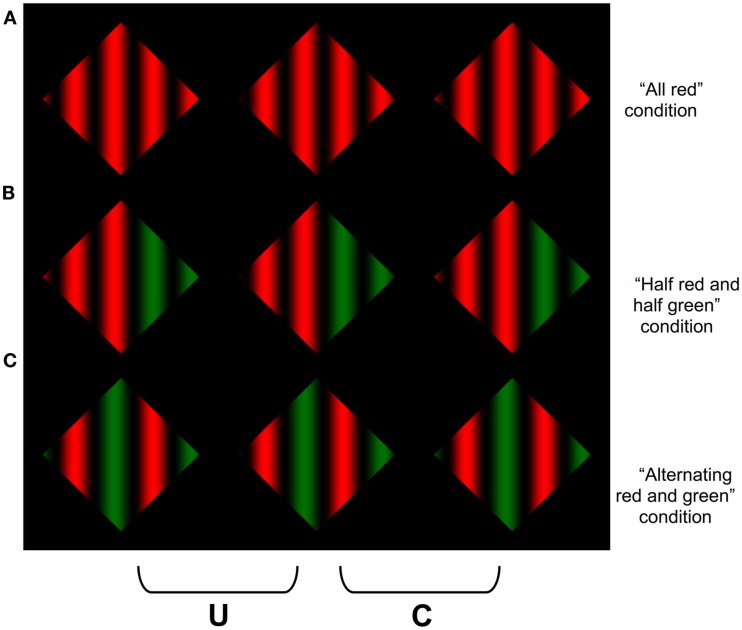
**A red-green variant of the displays used in Experiment 1, with an additional condition in which the color change occurred only once in the middle of the display**. When the two left columns are divergently fused, or the two right columns cross fused, coherent scission may be experienced in both **(A,B)**, but less so in **(C)**.

Our final experiments were designed to further explore the possibility that the difference in stereoscopic sensitivity to the gratings that contained differences in color were due to the difference in luminance between the different chromatic components. In Experiment 3, we tested whether a similar reduction in sensitivity would be observed for achromatic gratings that contained variations in the range of luminance values that occurred between alternating cycles (see Figure 8). Note that in these conditions, a coherent percept of transparency is still possible, since the lower contrast sinusoidal modulations could appear more opaque than the full contrast cycles of the sinusoid. In Experiment 4, we replicated the conditions of Experiment 1 using red and green colors that were approximately matched in luminance. These colors are also less prone to effects of chromatic aberration, since they are closer in physical wavelength composition. We added a control display to test whether it was the color difference *per se* that is responsible for the poorer sensitivity to disparity in the multi-colored displays. If the poorer performance in the multi-colored display arises from the difficulty in amodally completing the far surface, then this difference should be diminished if only a single color change is needed to perform the completion (see Figure 9).

## Experiments 3 and 4

### Participants

Two of the observers (AS and KT) who participated in Experiment 1 served as observers in Experiment 3. The same three observers who participated in Experiment 1 participated in Experiment 4.

### Apparatus and stimuli

For Experiment 3, stimuli were similar to those in Experiment 1, except the targets were now achromatic (Figure 8). The black-red modulations in the “all red” condition were replaced by sinusoidal modulations that varied the full luminance range of the monitor between “black” and “white” (with a maximum luminance of 98.365 cd/m^2^). The achromatic analog of the alternating red and blue were replaced by “white” and “light gray” (37.930 cd/m^2^), respectively (Figure 8, bottom).

For Experiment 4, stimuli were similar to those in Experiment 1, except the blue modulations were replaced with green, and the red and green were approximately matched in luminance (Figure 9). A third condition (“half red and half green”) was also introduced, in which the left half of the target contained red-black sinusoidal modulations and the right contained green-black modulations (Figure 9, middle). The luminance of the maximal green was 24.52 cd/m^2^ (CIE *xy*Y coordinates *x* = 0.33, *y* = 0.63), and the luminance of the maximal red was 22.308 cd/m^2^ (CIE *xy*Y coordinates *x* = 0.65, *y* = 0.34).

### Procedure

The procedure in Experiments 3 and 4 was identical to that of Experiment 1.

## Results and Discussion

**Figure 10:**
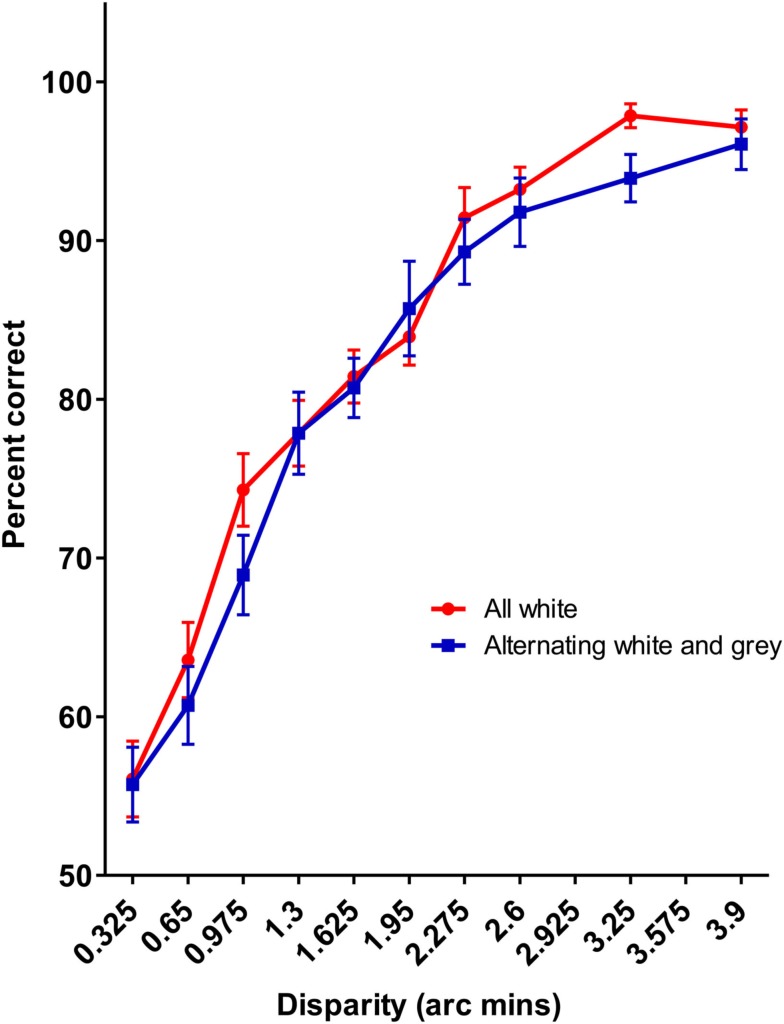
**The results of Experiment 3**. No difference was observed for the achromatic mixture of amplitudes and the full contrast sinusoidal pattern.

The results of Experiment 3 are presented in Figure 10. No difference in sensitivity between the uniform sinusoidal luminance cycles and the varying amplitude cycles is observed (*p* = 0.411), which suggests that it was not the luminance difference between the different colors that is responsible for the reduction in sensitivity to the alternating red-blue gratings used in Experiment 1.

**Figure 11:**
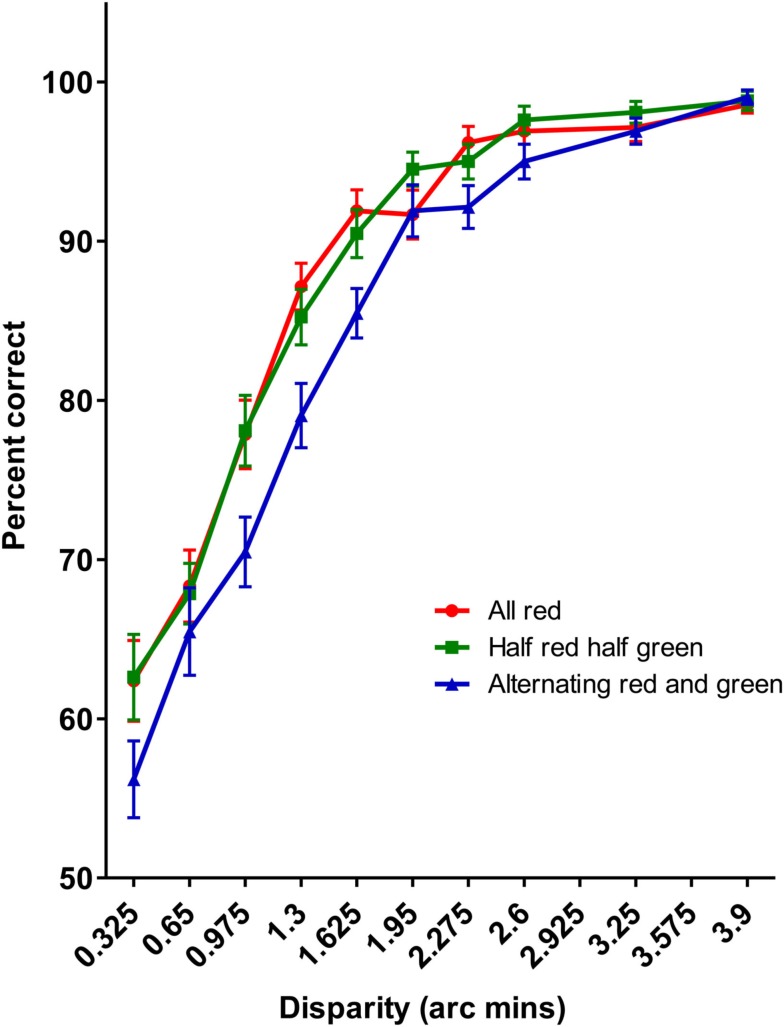
**The results of Experiment 4**. As with Experiment 1, it was significantly harder detecting disparity increments in the alternating red-green display than either the uniformly red or half-red/half-green displays.

This conclusion is also supported by the results of Experiment 4 (see Figure 11). As in Experiment 1, sensitivity to disparity increments in the display containing the alternating modulations of red and green was worse that either the uniformly colored display (*p* = 0.008) or the display in which the colors were divided along the midline of the display, despite being approximately matched for luminance. There was no significant difference between the all red condition and the half-red/half-green condition (*p* = 0.292). These data replicate the results of Experiment 1 using different, luminance matched colors, suggesting that it is the spatial variation in colors that is responsible for the reduced sensitive to disparity increments in these displays.

## General Discussion

The preceding experiments were designed to assess whether ability to scission stereoscopic textures into layered image representations is limited by constraints on amodal surface completion. The striking aspect of the stereo displays used in the present studies, as well as those used previously, is that the textures (gratings) contain only a single value of disparity within the texture. The percept of scission, when it occurs, must arise from information derived from the far disparities that arise along the aperture boundary. Previous work using achromatic displays focused on the role of (luminance) contrast polarity in predicting when transparency can or cannot be obtained in such displays (Anderson, 1999, 2003a; Anderson et al., 2002). All of the conditions that have been shown to elicit global percepts of transparency and occlusion in these textures have been consistent with, and generated percepts of, a uniformly colored, amodally completed surface. The present experiments were designed to assess whether the depth segregation that arises in these displays depends on the ability to form a uniformly colored amodal surface. Our experiments suggest that it is indeed harder to detect disparity increments for displays that require amodally completing surfaces that involve integrating multiple surface color changes. Note that the formation of an amodally completed surface in these color change displays could only occur if the surface regions assigned to the near disparity in these displays completely occluded the color transitions of the underlying surface. Our data suggest that the improbability of such events limits the capacity of observers to detect the disparity differences in these displays, which suggests that such surface level computations can limit the sensitivity of simple disparity based judgments.

**Figure 12:**
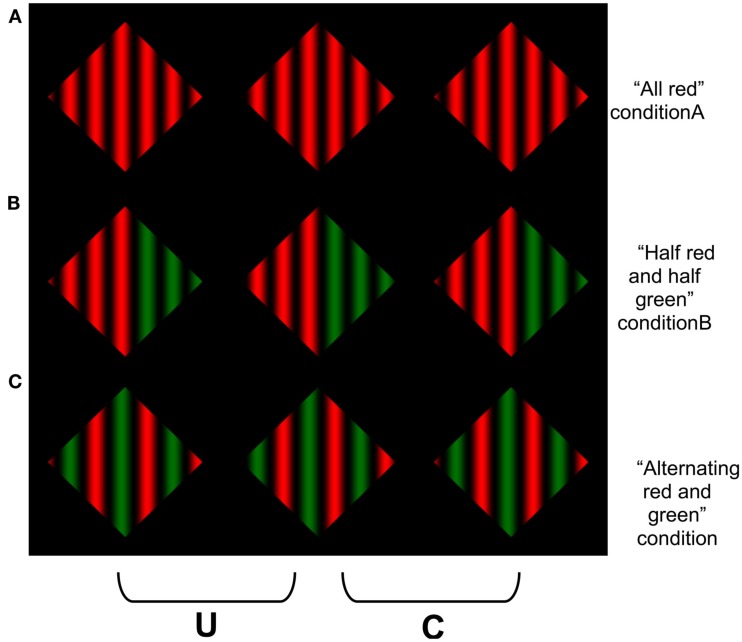
**Demonstration of the difficulty in amodally completing the displays containing more numerous changes in the amodally completed surface color**. Observers report that it is harder to obtain a clear percept of an amodally completely surface in **(C)** than in **(A)** or **(B)**. This image is similar to Figures 4 and 9, but uses a higher spatial frequency. The difference between the alternating cycle display than the other display types is more pronounced as the spatial frequency increases. To avoid the modal completion of the dark “components” of the grating across the surround, the reader is advised to occlude the other images in the figure when viewing each row of the figure.

Our hypothesis also receives strong phenomenological support by simply fusing the stereograms containing alternating color cycles, and comparing their perceptual coherence with those composed of a single color or requiring only a single color change. Due to the periodic structure of our stimuli, we used relatively low frequency gratings in our experiments to avoid false matches that can arise from the well-known stereoscopic “wallpaper effect.” However, the differences between the alternating color gratings and the uniform or single color changes become quite striking when the number of cycles are increased (see Figure 12). The gratings in Figures 12A,B both appear coherently segmented into multiple layers, whereas it is extremely difficult to experience any sense of a coherent, global percept of scission for the alternating red-green color cycles in Figure 12C.

Our results provide further evidence for the intimate link between the segmentation processes responsible for the perceptual scission that occurs in conditions of transparency and occlusion, and the interpolation or linking processes that are involved in the formation of amodally completed surface structure (Anderson and Julesz, 1995; He and Ooi, 1998; Singh and Anderson, 2000; Anderson et al., 2002, 2009; Anderson, 2007). Our data demonstrate that the ability to sense disparity defined depth differences can be limited by processes involved in decomposing images into multiple layers, and integrating the components of each layer into a coherent surface representation. It is not possible to assess the role of amodal completion in modulating when scission does or does not occur in the kinds of stereoscopic textures used herein using purely achromatic displays because variations in achromatic color are confounded with polarity constraints. However, such confounds can be overcome by modulating both the chromatic content and luminance of our textures simultaneously. From a methodological perspective, our results suggest that color, in addition to being an important topic of surface perception in its own right, can be used as a tool to understand the kinds of processes that underlie achromatic surface computations (Anderson et al., 2011).

It should be noted that many of the effects in the experiments reported herein can be experienced monocularly as well as stereoscopically, and hence, should not be construed as stereoscopic phenomena *per se*. Indeed, the CDAP and the TAP express general conditional constraints about how the perception of lightness, depth, and transparency can be derived from the pattern of relative luminance in images. Our colored stimuli also contained variations in luminance, and hence, are fully within the predictive scope of the CDAP and TAP. Future research is needed to determine whether these principles can be generalized to purely chromatic forms of transparency, which admit a broader range of image transformations than achromatic transparency (D’Zmura et al., 2000; Ekroll et al., 2002; Faul and Ekroll, 2002, 2011; Khang and Zaidi, 2002; Wollschläger and Anderson, 2009).

## Conflict of Interest Statement

The authors declare that the research was conducted in the absence of any commercial or financial relationships that could be construed as a potential conflict of interest.
